# DeepStrain: A Deep Learning Workflow for the Automated Characterization of Cardiac Mechanics

**DOI:** 10.3389/fcvm.2021.730316

**Published:** 2021-09-03

**Authors:** Manuel A. Morales, Maaike van den Boomen, Christopher Nguyen, Jayashree Kalpathy-Cramer, Bruce R. Rosen, Collin M. Stultz, David Izquierdo-Garcia, Ciprian Catana

**Affiliations:** ^1^Department of Radiology, Athinoula A. Martinos Center for Biomedical Imaging, Massachusetts General Hospital and Harvard Medical School, Boston, MA, United States; ^2^Harvard-MIT Division of Health Sciences and Technology, Cambridge, MA, United States; ^3^Department of Radiology, University Medical Center Groningen, University of Groningen, Groningen, Netherlands; ^4^Cardiovascular Research Center, Massachusetts General Hospital and Harvard Medical School, Boston, MA, United States; ^5^Department of Electrical Engineering and Computer Science, Massachusetts Institute of Technology, Cambridge, MA, United States; ^6^Division of Cardiology, Massachusetts General Hospital, Boston, MA, United States

**Keywords:** cine-MRI, deep learning, segmentation, motion estimation, myocardial strain

## Abstract

Myocardial strain analysis from cinematic magnetic resonance imaging (cine-MRI) data provides a more thorough characterization of cardiac mechanics than volumetric parameters such as left-ventricular ejection fraction, but sources of variation including segmentation and motion estimation have limited its wider clinical use. We designed and validated a fast, fully-automatic deep learning (DL) workflow to generate both volumetric parameters and strain measures from cine-MRI data consisting of segmentation and motion estimation convolutional neural networks. The final motion network design, loss function, and associated hyperparameters are the result of a thorough *ad hoc* implementation that we carefully planned specific for strain quantification, tested, and compared to other potential alternatives. The optimal configuration was trained using healthy and cardiovascular disease (CVD) subjects (*n* = 150). DL-based volumetric parameters were correlated (>0.98) and without significant bias relative to parameters derived from manual segmentations in 50 healthy and CVD test subjects. Compared to landmarks manually-tracked on tagging-MRI images from 15 healthy subjects, landmark deformation using DL-based motion estimates from paired cine-MRI data resulted in an end-point-error of 2.9 ± 1.5 mm. Measures of end-systolic global strain from these cine-MRI data showed no significant biases relative to a tagging-MRI reference method. On 10 healthy subjects, intraclass correlation coefficient for intra-scanner repeatability was good to excellent (>0.75) for all global measures and most polar map segments. In conclusion, we developed and evaluated the first end-to-end learning-based workflow for automated strain analysis from cine-MRI data to quantitatively characterize cardiac mechanics of healthy and CVD subjects.

## Introduction

Cardiac mechanics reflects the precise interplay between myocardial architecture and loading conditions that is essential for sustaining the blood pumping function of the heart. The ejection fraction (EF) is often used as a left-ventricular (LV) functional index, but its value is limited when mechanical impairment occurs without an EF reduction (1). Alternatively, tissue tracking approaches for strain analysis provide a more thorough characterization through non-invasive evaluation of myocardial deformation from echocardiography or cinematic magnetic resonance imaging (cine-MRI) data (2), and could be used to identify dysfunction before EF is reduced (3). Unfortunately, various sources of discrepancies have limited the wider clinical applicability of these techniques, including factors related to imaging modality, algorithm, and operator (4). More accurate measures could be obtained from tagging-MRI data widely regarded as the reference standard for strain quantification (5, 6), but use of these data is less common partly due to lack of available analysis tools, whereas echocardiography and cine-MRI data are ubiquitously acquired and analyzed in clinical practice.

Irrespective of algorithm or modality, e.g., speckle tracking for echocardiography or feature tracking for cine-MRI, the main challenge is to estimate motion within regions along the myocardial wall (2). Operator-related discrepancies are introduced when the myocardial wall borders are delineated manually, a time-consuming process that requires considerable expertise and results in significant inter- and intra-observer variability (7, 8). Automatic delineation approaches have been implemented within computational pipelines (9), but other factors related to motion tracking algorithms also influence strain assessment, including the appropriate selection of tuneable parameters whose optimal values can differ between patient cohorts and acquisition protocols [e.g., the size of the search region in block-matching methods (10)]. Further, these algorithms often make assumptions about the properties of the myocardial tissue [e.g., incompressible and elastic (11, 12)], or use registration methods to drive the solution toward an expected geometry. However, recent evidence has shown the validity of these assumptions varies between healthy and diseased myocardium (13, 14), suggesting these approaches may not accurately reflect the underlying biomechanical motion. Modality-related image quality could also complicate interpretation of abnormal strain values since these could reflect either real dysfunction or artifact-related inaccuracies, leading to some degree of subjectivity or non-conclusive results (3). Lastly, although automated segmentation and motion tracking commercial software is available for cardiac cine imaging, manual correction of delineated contours used for tracking is often required, resulting in significant variations in strain depending on segmentation procedure and type of commercial software (15).

Deep Learning (DL) methods have demonstrated the advantage of allowing real-world data guide learning of abstract representations that can be used to accomplish pre-specified tasks, and have been shown to be more robust to image artifacts than non-learning techniques for some applications (16, 17). DL segmentation methods have been proposed (18–21) and implemented within strain computational pipelines (22, 23), and recent studies have shown that cardiac motion estimation can also be recast as a learnable problem (24–28). These methods usually consist of an intensity-based loss function and a constrain term (24, 27), the latter using common machine learning techniques [e.g., L2 regularization of all learnable parameters (25)] or direct regularization of the motion estimates [e.g., smoothness penalty (24), anatomy-aware (28)]. However, none of these methods have considered the accuracy of myocardial strain as a design factor or have been applied to strain analysis.

We have recently developed a learning-based method for cardiac motion estimation that produces more accurate estimates than various techniques, including B-spline, diffeomorphic, and mass-preserving algorithms (29), and showed these estimates could potentially be used to detect regional dysfunction. Thus, incorporating our method within a strain analysis framework could potentially enable accurate, user-independent, and quantitative characterization of cardiac mechanics at a both global and regional level. While this framework could be based on echocardiography images (30), these data remain limited for strain mapping tasks by their low reproducibility of acquisition planes (4) and temporal stability of tracking patterns (31). In contrast, cine-MRI offers the most accurate and reproducible assessment of cardiac anatomy and function, thus providing a more thorough set of data for learning-based motion models.

We propose DeepStrain, a fast, automated workflow that derives global and regional strain measures from cine-MRI data by decoupling motion estimation and segmentation tasks. With decoupling, segmentations are not used for motion estimation during inference but rather to derive clinical parameters and to identify a cardiac coordinate system for strain analysis, further reducing the variability in strain directly related to segmentation. Although two-dimensional (2D) convolutional neural networks (CNN) for cardiac motion estimation from cine-MRI have been proposed (24, 26, 28, 32), DeepStrain is the first end-to-end learning based workflow for myocardial strain analysis from cine-MRI. In addition, motion predicted using 2D architectures could be influenced by out-of-plane motion during the cardiac cycle, resulting in overestimation of in-plane motion and reduced reproducibility (33). Instead, this paper describes a carefully designed strain quantification-specific 3D CNN that handles challenges associated with the anisotropic resolution of cine-MRI data. Our loss weighting strategy to find the optimal balance between motion regularization terms also differs from previous methods which have traditionally relied on registration techniques as indirect measures of motion accuracy (24, 26, 28, 32). Instead, we simulated cine-MRI data with corresponding ground-truth cardiac motion to identify the hyperparameters yielding accurate motion and strain estimates. The optimal trained configuration is online at https://github.com/moralesq/DeepStrain. Finally, this paper also provides a comprehensive assessment of the accuracy and repeatability of DeepStrain measures, a task that has been mostly ignored in the deep learning literature but is critical to clinical adoption (4).

## Methods

### Myocardial Strain Definitions

Strain represents percent change in myocardial length per unit length. The 3D analog for MRI is given by the Green-Lagrange strain tensor

(1) $$E\left(t\right)=\left(\nabla u\left(t\right)\;+\left(\nabla u\left(t\right)\right){\;}^{T}+{\left(\nabla u\left(t\right)\right)}^{T}\nabla u\left(t\right)\right)/2,$$

where ***u***(*t*) denotes myocardial displacement from a fully-relaxed end-diastolic (ED) phase at *t* = 0, to a contracted frame at *t* >0. Radial and circumferential strain are the diagonal components of the tensor **E** evaluated in cylindrical coordinates. *Strain rate (SR)* is the time derivative of (1). The time of acquisition of each frame was extracted from the DICOM and was used to interpolate **E**(t), such that **E**(t) was defined at every millisecond. The time derivative was then evaluated using central differences and reported as change in strain per second with unit s^−1^.

*Global strain* is defined as the average of **E** over the whole LV myocardium (LVM) volume. *Regional* strain is defined as the average of **E** over the volume of specific LVM segments defined by the American Heart Association (AHA) polar map (34), which requires labels of the right ventricle to construct. Specific parameters based on timing and magnitude are extracted from the measures evaluated over a whole cardiac cycle: *end-systolic strain (ESS)*, defined as the global strain value at end-systole (ES); *systolic strain rate (SRs)*, defined as the peak (i.e., maximum) absolute value of global SR during systole; *early-diastolic strain rate (SRe)*, defined as the peak absolute value of global SR during diastole. Although only radial and circumferential strain were analyzed in this study, DeepStrain is also capable of generating shear (Supplementary Section 1). The code used to construct the AHA polar maps is available in the repo online.

### Centering, Segmentation, and Motion Estimation

**Figure 1:**
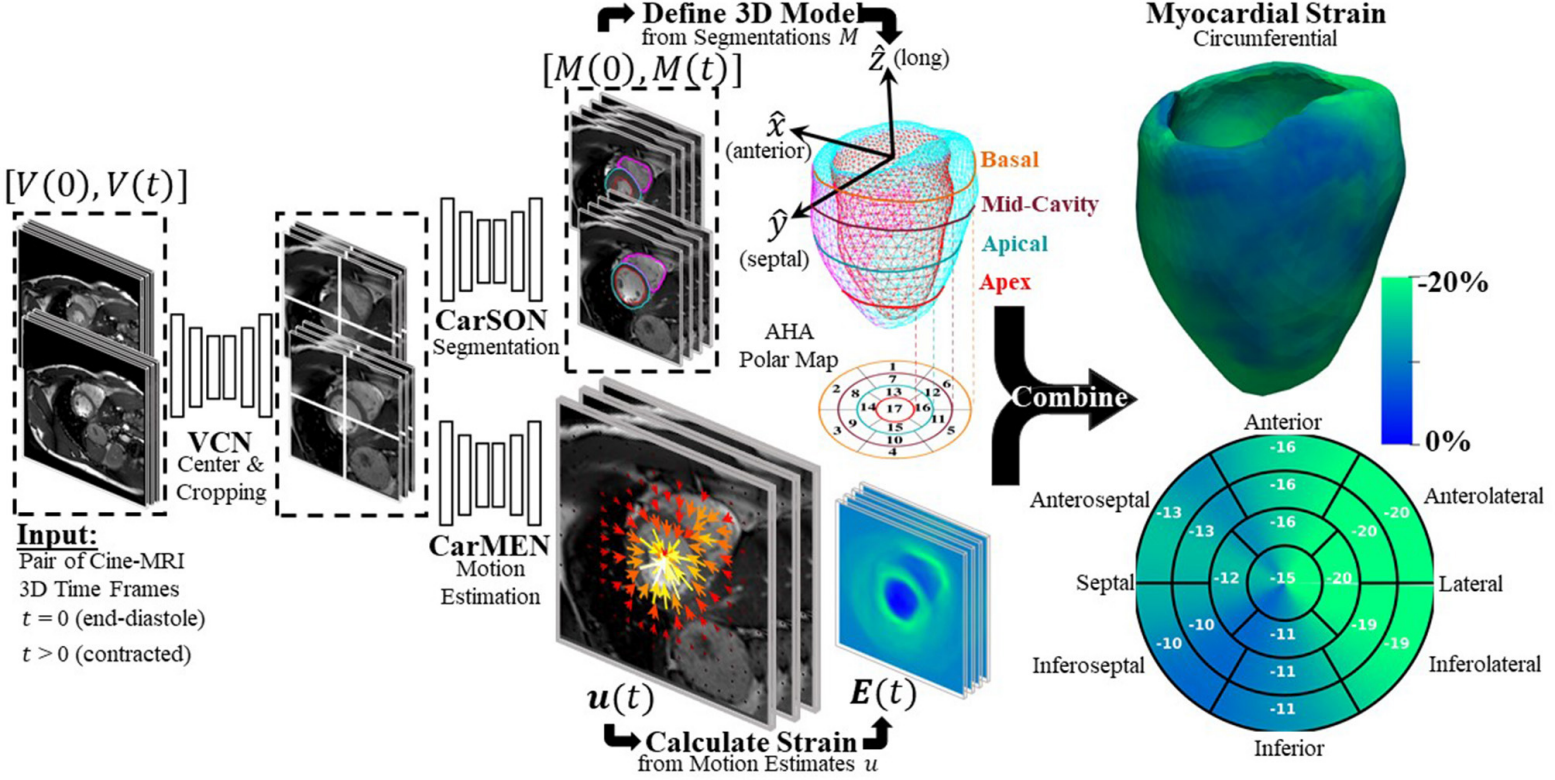
Overview of proposed DeepStrain workflow. VCN centers and crops the input pair of cine-MRI frames. Tissue labels generated by CarSON are used to build an anatomical model. Motion estimates derived from CarMEN are used to calculate strain measures, and these estimates are combined with the anatomical model to enable global and regional strain analyses.

DeepStrain (Figure 1) consists of a series of CNNs that perform three tasks: a ventricular centering network (VCN) for automated centering and cropping, a cardiac segmentation network (CarSON) to generate tissue labels, and a cardiac motion estimation network (CarMEN) to generate ***u***. Estimates of ***u*** are used to calculate myocardial strain, and segmentations are used to derive volumetric parameters, identify a cardiac coordinate system for strain analysis, and generate tissue labels used for anatomical regularization of motion estimates at training time.

All networks have a common encoder-decoder architecture consisting primarily of convolution, batch normalization (35), and PReLU (36) layers with residual connections (37). The specific architecture formulation and losses are discussed below and Supplementary Section 2.

#### VCN

Let *V*_*t*_ be a cine-MRI frame at time *t* defined over a *n*-D domain Ω⊂ℝ^*n*^, and let *v* ∈Ω. VCN uses a single-channel array *V* with size 256 × 256 × 16 to generate a single-channel array *G*_*pred*_ of equal size, where *G*_*pred*_ corresponds to a Gaussian distribution with mean defined as the LVM center of mass. This approach models the uncertainty associated with the center location, specially in pathological cases, and enables automated generation of ground-truth labels when manual segmentation of uncropped images is available. VCN was trained using the mean square error (MSE) loss function

(2) $$L_{MSE}\left(G_{gt},G_{pred}\right)=\frac{1}{\left|\Omega \right|}\sum_{v\in \Omega }{\left(G(v)-G_{pred}\left(v\right)\right)}^{2},$$

where *G*_*gt*_ is the ground-truth Gaussian distribution. At inference, the input volume *V* is centered and cropped around the voxel with the highest value in *G*_*pred*_ to generate a new cropped array of size 128 × 128 × 16, which is then the input to CarSON and CarMEN.

#### CarSON

CarSON is a 2D architecture that uses single-channel images *V* of size 128 × 128 to generate a 4-channel segmentation *M*_*pred*_ of equal size, each channel corresponding to a label. We experimented with two different loss functions $L_{seg}$ to train CarSON using the manual segmentations *M*_*ms*_: the pixel-wise categorical cross-entropy (CCE), and a multi-class Dice coefficient (MDC) loss function

(3) $$L_{MDC}\left(M_{ms},M_{pred}\right)=-\frac{1}{K}\sum_{k=0}^{3}2\frac{\left|v_{ms}^{k}\cap v_{pred}^{k}\right|}{\left|v_{ms}^{k}\right|+\left|v_{pred}^{k}\right|},$$

where *k*∈ [0, 3] represents each of the tissue labels (i.e., background, RV, LVM, and LV), and *v*^*k*^∈*M* denotes all the pixels with label *k*.

#### CarMEN

CarMEN estimates the motion ***u***_*t*_ of the heart from *V*_0_ to *V*_*t*_, i.e., for each voxel *v*∈Ω, ***u***_*t*_(*v*) is an approximation of the myocardial displacement during contraction such that *V*_0_(*v*) and (***u***_*t*_°*V*_*t*_)(*v*) correspond to similar cardiac regions. The operator ° refers to application of a spatial transform to *V*_*t*_ using ***u***_*t*_ via trilinear interpolation (38). Thus, CarMEN uses a 2-channel input volume consisting of two concatenated arrays with size 128 × 128 × 16 to generate a 3-channel array ***u*** of equal size, each channel representing the *x*, *y*, and *z* components of motion.

Although the current formulation of CarMEN shares some similarities with our previous work, we have made several design modifications that were specific for accurate strain quantification. Here a combination of three loss functions was used for training: first, we used an unsupervised loss function $L_{intensity}$ that trains CarMEN using the input volumes and generated motion estimates

(4) $$L_{intensity}\left(V_{0},V_{t},u_{t}\right)=\frac{1}{\left|\Omega \right|}\sum_{v\in \Omega }\left|\left(V_{0}\left(v\right)-(u_{t}{}^{\circ}V_{t}\right)\left(v\right)\right|.$$

Second, we used a supervised function $L_{anatomical}$ that leverages segmentations of the input volumes at training time to impose an anatomical constrain on the estimates

(5) $$L_{anatomical}\left(M_{0},\;M_{t},u_{t}\right)=\;L_{seg}\left(M_{0},u_{t}{}^{\circ}M_{t}\right).$$

Third, smooth estimates were encouraged by using a diffusion regularizer

(6) $$L_{smoothness}(u_{t})={\sum_{v\in \Omega }\left\|\nabla u_{t}\left(v\right)\cdot dr\right\|}^{2}$$

where *dr* is the spatial resolution of *V*. Thus, the loss function for CarMEN is a linear combination of (4), (5), and (6), weighted by λ_*i*_, λ_*a*_, λ_*s*_, accordingly.

Some design variations were exclusive to estimation of motion from 3D cine-MRI frames. Convolution, pooling, and upscaling was implemented with 3 × 3 × *k*_*z*_ operations, where *k*_*z*_ could be set to either 1 or 3. For *k*_*z*_ = 1, operations were carried out only in the x-y-plane to account for the low and varying z-resolution, different from 3D architectures for segmentation with 3 × 3 × 3 convolutions and in-plane-only pooling and upscaling (39). Thus, context in the z-dimension is aggregated through trilinear interpolation of *V*_*t*_ and *M*_*t*_ volumes in (4) and (5), and through application of 3D spatial gradients to *u* in (6). The spatial gradient in (6) also includes an additional term *dr* to account for differences between in-plane and slice resolution which was not used in (40). Lastly, we experimented with CCE and MDC implementations as anatomical constrains in (5).

At inference, the entire cycle of a single subject can be analyzed using sequential inputs

$$\begin{array}{l} {\left\{\left(V_{0},\;V_{t}\right)\right\}}_{\left\{t=0,1,\ldots ,T\right\}}\text{ to derive }{\left\{u_{t}\right\}}_{\left\{t=\;0,1,\ldots ,T\right\}}. \end{array}$$

## Experiments

### Datasets

For development we used the *Automated Cardiac Diagnosis Challenge (ACDC)* dataset (41), consisting of cine-MRI data from 150 subjects evenly divided into five groups: healthy and patients with hypertrophic cardiomyopathy (HCM), abnormal right ventricle (ARV), myocardial infarction with reduced ejection fraction (MI), and dilated cardiomyopathy (DCM). These data were publicly available as train (*n* = 100) and test (*n* = 50) sets, with manual segmentations included for the train set only. For validation of motion and strain measures we used the *Cardiac Motion Analysis Challenge (CMAC)* dataset (42), consisting of paired tagging- and cine-MRI data from 15 healthy subjects. To assess intra-scanner repeatability, 10 healthy volunteers were recruited to undergo repeated scans on a 3T MRI scanner (Supplementary Section 3). All cine-MRI frames and corresponding segmentations were resampled to a 256 × 256 × 16 volume grid with 1.25 mm × 1.25 mm in-plane resolution and variable slice thickness (4–7 mm).

### DeepStrain Implementation

For optimization experiments and final model training, all networks were trained in TensorFlow ver. 2.0 with Adam optimizer parameters beta 1, 2 = 0.9, 0.999, random initialization, batchsize = 80 (5 for CarMEN), and learning rate = 1e-4.

#### Design of a Strain Quantification-Specific CNN

**Figure 2:**
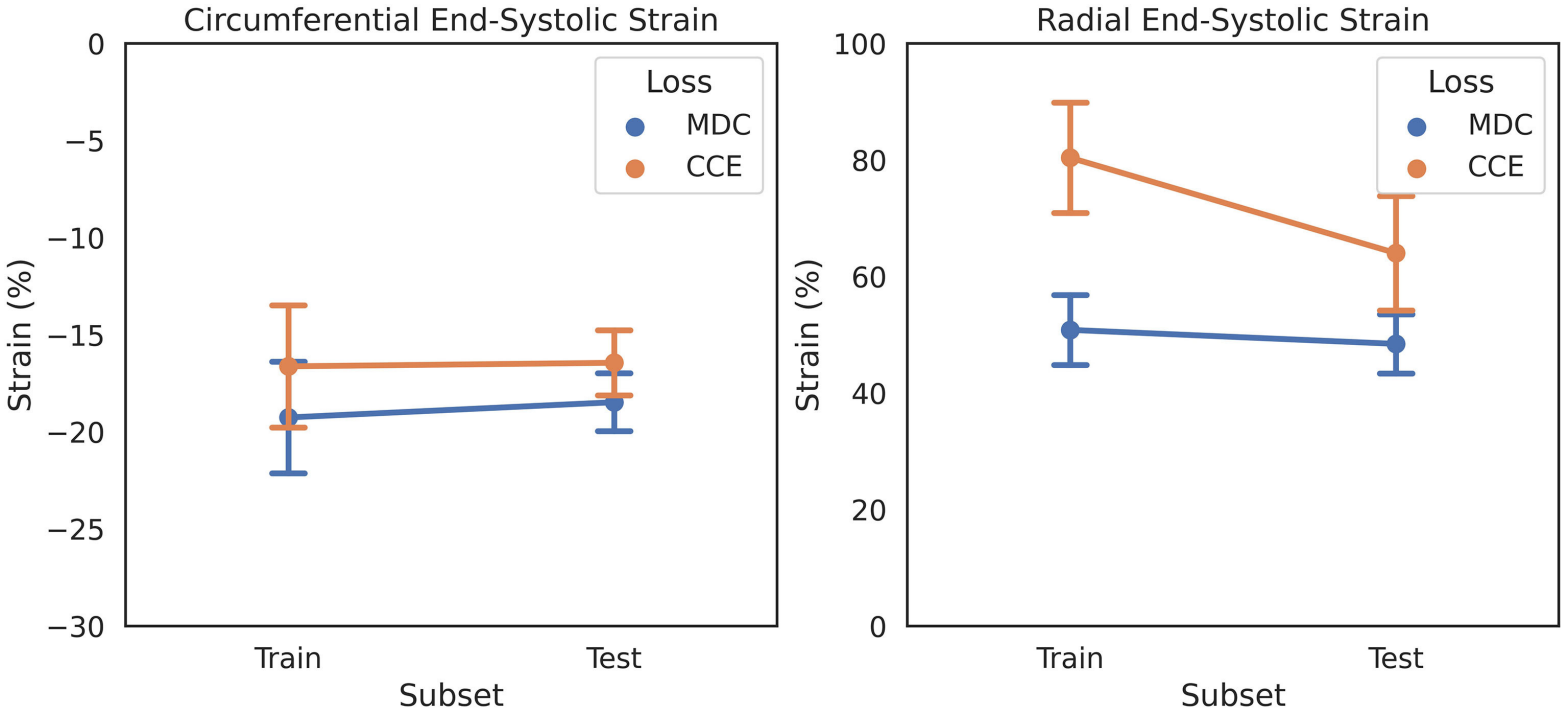
Effect of anatomical regularization of motion estimates on strain on the ADCD dataset. Regularization with multiclass dice coefficient (MDC) and categorical crossentropy (CCE) functions result in different strain values in healthy subjects, shown as mean and standard deviation.

Reported normal ranges of strain in healthy individuals using non-learning methods vary largely between the different deformation methodologies, limiting the clinical utility of strain measures (4). We used this concept as a heuristic in updating CarMEN, i.e., a useful design should minimize the variation in strain values in healthy individuals. To assess the impact of design choices on this heuristic, we separated the ACDC training set into two group-balanced train and test subsets, each with 50 subjects. We trained CarMEN for 300 epochs using two different layer operation sizes (i.e., 3 × 3 × *k*_*z*_ with *k*_*z*_∈{1, 3}), and two different implementations of (5) (i.e., MDC and CCE). With *k*_*z*_ = 3, comparison of losses showed that CCE leads to increased standard deviation in radial ESS in healthy train (*n* = 10) and test (*n* = 10) subjects, and large differences in the average radial ESS between training and testing sets (Figure 2). Multiple experiments with different regularization parameters showed similar results, and showed that setting *k*_*z*_ = 1 reduces deviations in healthy strain (Supplementary Table 1). Thus, the new CarMEN design used 3 × 3 × 1 operations and was regularized using the MDC function.

#### Novel Loss Weighting Strategy for Accurate Motion and Strain Estimation

**Figure 3:**
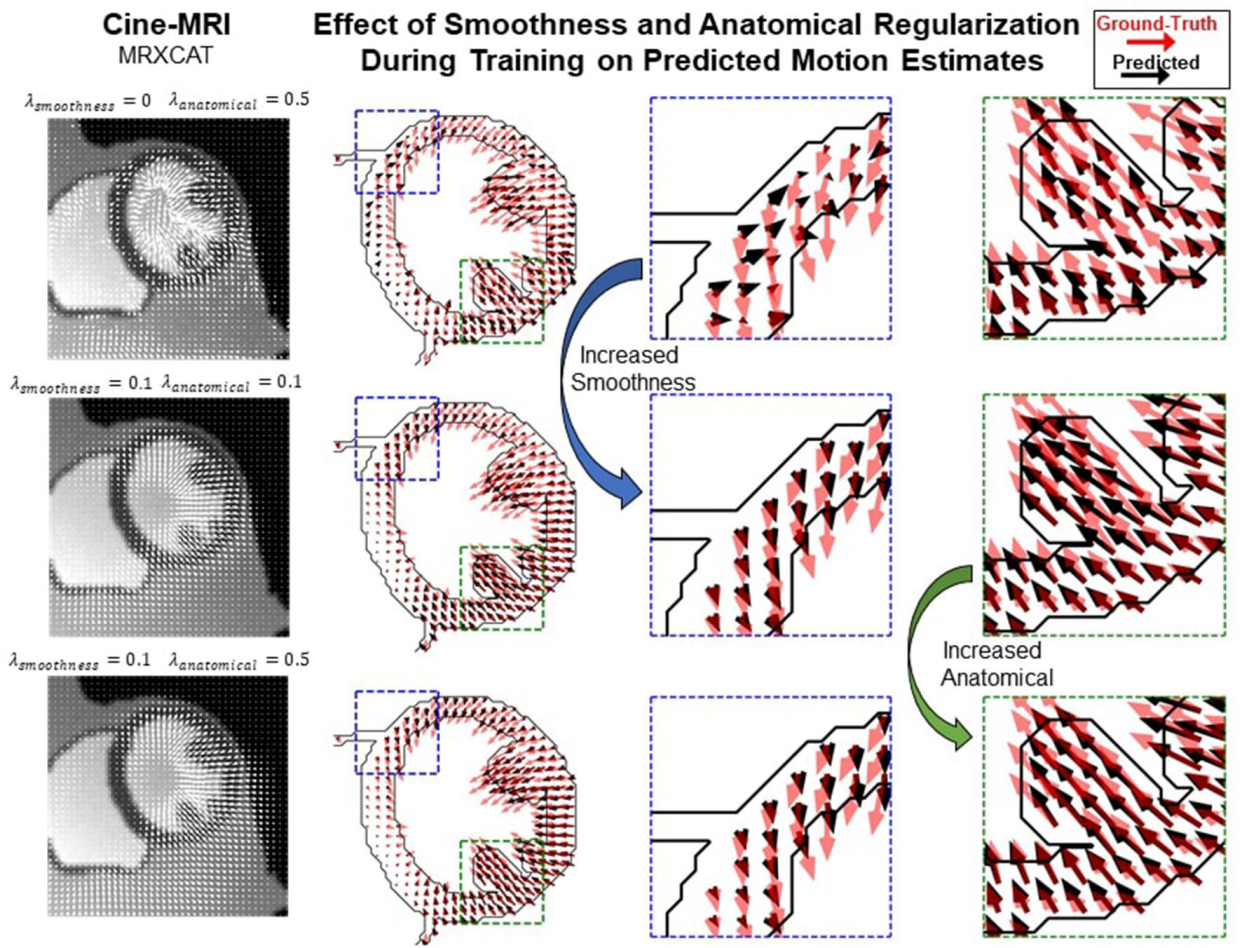
Qualitative effects of smoothing and anatomical regularization on the accuracy of motion estimates on the MRXCAT dataset. First row shows the predicted (black) motion estimates when the anatomical regularization is set to 0.5 and smoothing is set to 0. Relative to the ground-truth (red), these estimates are highly irregular. Increasing (third column) the smoothness to 0.1 and setting anatomical to 0.1 improves the direction of the estimates, but the magnitude is reduced. This is corrected by increasing anatomical regularization to 0.5 (fourth column).

**Figure 4:**
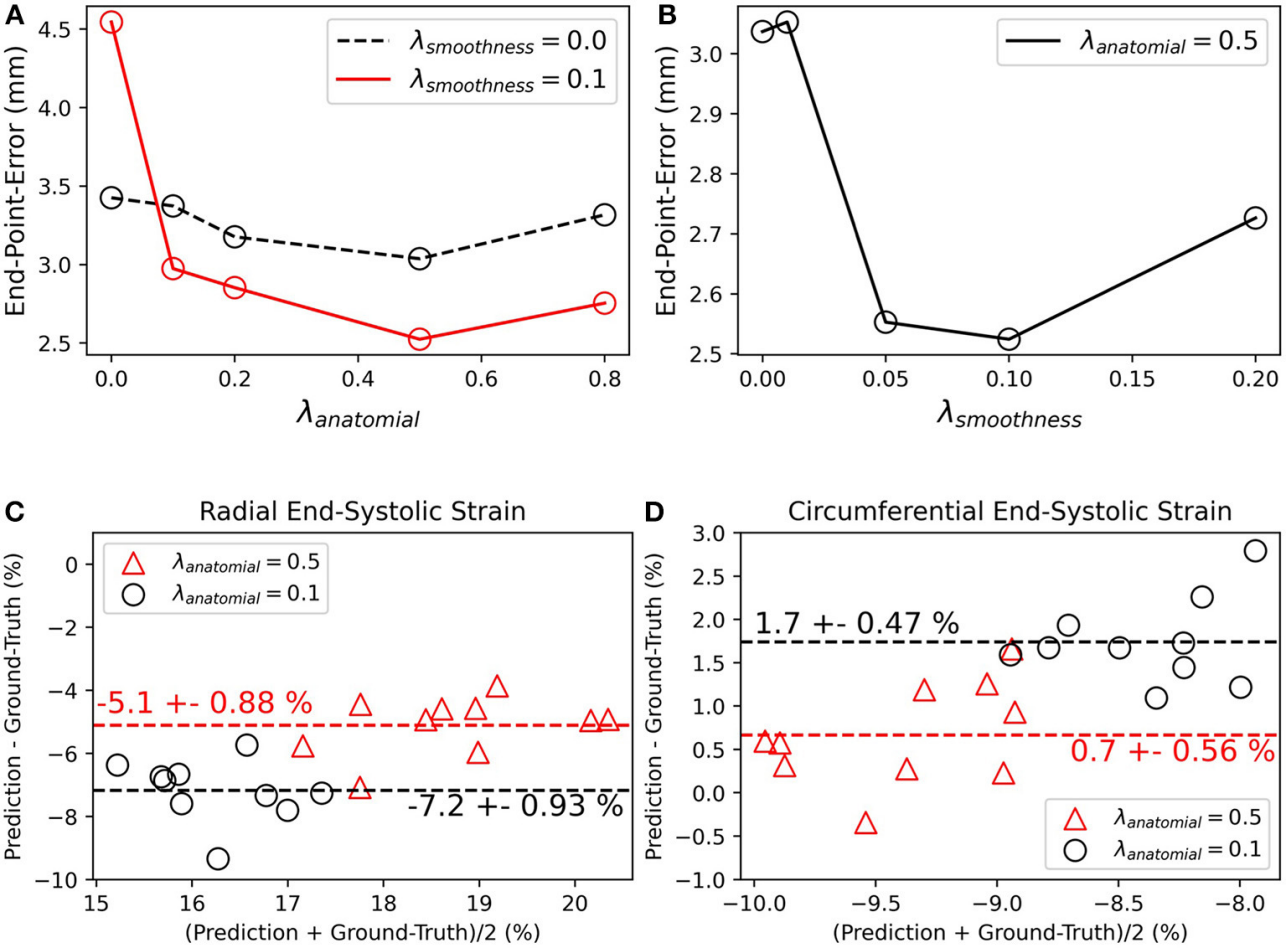
Quantitative effects of regularization on the accuracy of motion and myocardial strain. **(A,B)** End-point-error on MRXCAT test data with ground-truth motion for CarMEN with varied **(A)** anatomical and **(B)** smoothing regularization parameters. **(C,D)** Bland-Altman plots of radial and circumferential end-systolic strain for two different anatomical regularization parameters, and smoothing set to 0.1.

Most proposed networks to-date have used registration terms such as (4) and (5) to indirectly assess the accuracy of *u*_*t*_ on validation or test datasets. However, this approach is prone to errors since inaccurate and even unrealistic *u*_*t*_ solutions can minimize these terms. To find an optimal balance between loss terms, we simulated 10 cardiac cine-MRI frames at ED and ES with known ground-truth motion using the MR-extended cardiac-torso (MRXCAT) (43, 44), a software phantom used extensively in imaging studies (45). The motion of the software phantom was modeled using gated patient 4D tagging data, producing highly realistic contracting and twisting motion of the normal heart that can be parameterized to generate population-wide characteristics, as previously described by us (29). We trained CarMEN with various regularization parameters for 300 epochs using 100 subjects from the ACDC training set, and tested the models on the MRXCAT data by evaluating the end-point error between ground-truth and predicted motion estimates within the LVM (Figure 3). Setting λ_*s*_ = 0 leads to highly irregular motion vectors (e.g., off by more than 90 degrees) relative to ground-truth. Setting the smoothness and anatomical weights to λ_*s*_ = λ_*a*_ = 0.1 leads to smoother and better aligned vectors, albeit with a slightly decreased magnitude. Increasing the anatomical weight to λ_*a*_ = 0.5 further improves the estimates by generating vectors with similar magnitude and orientation to the ground-truth. Quantitative measures of motion accuracy showed similar results across various regularization values, and these changes in motion estimation accuracy were reflected as bias changes in strain values (Figure 4). We found the optimal parameters to be λ_*i*_ = 0.01, λ_*a*_ = 0.5, λ_*s*_ = 0.1, which in addition resulted in low strain deviation in healthy subjects as described in the previous section (Supplementary Table 1). Thus, the optimal architecture and hyperparameters were selected based on both the ACDC (i.e., to assess strain deviation in healthy subjects) and XCAT (i.e., to assess motion and strain accuracy).

#### Final Model Training

Ground-truth distributions for VCN were created using the manual segmentations. VCN and CarSON were trained using the ED and ES frames of the train set, as only these included ground-truth segmentations. This provided 200 training samples for VCN and 3200 for CarSON, the latter having more samples since it is a 2D architecture and all frames were resampled to a volume with 16 slices. VCN was tested by five-fold cross-validation, whereas the accuracy of CarSON was assessed by submitting the results to the challenge website. Once CarSON was trained, we generated segmentations of the test set to train CarMEN using the entire ACDC dataset, i.e., 100 subjects from the train set with manual segmentations and 50 from the test set with CarSON-predicted segmentations. Only the ED-ED and ED-ES pairs were used for training. The former pair is useful for the network to learn the identity transformation. Data augmentation included random rotations and translations, random mirroring along the x and y axes, and gamma contrast correction. All data augmentation was performed only in the x-y plane.

### Evaluation Metrics

#### Segmentation and Motion Estimation

The CarSON-predicted and manual segmentations were compared using the Hausdorff distance (HD) and Dice Similarity Coefficient (DSC) metrics at both ED and ES. Accuracy of LV volumetric measures derived from segmentations, including ED volume (EDV), EF, and LVM, was assessed using the correlation, bias, and standard deviation metrics. The mean absolute error (MAE) for the LV EDV and LVM were also computed for comparison against the intra- and inter-observer variability reported by (41). RV labels were not analyzed since they were not used to assess cardiac function but rather to define the direction of the septal wall, which is needed to construct the LV strain polar maps with a normalize orientation between subjects. We compared our results to top-3 ranked methods published for the ACDC test set as these appear in the leader-board of the challenge (18, 20, 21, 39).

CMAC organizers defined 12 landmarks at intersections of gridded lines on tagging images at ED, one landmark *p*_0_ per wall (septal, inferior, lateral, interior) per ventricular level (basal, mid, septal). These landmarks were manually-tracked on tagging images by two observers over the cardiac cycle, and each position was transformed from tagging to cine coordinates using DICOM header information. We used the CarMEN motion estimates *u*_*t*_ to automatically deform the landmarks at ED, and the accuracy was assessed using the in-plane end-point error (EPE) between deformed $p_{t}{}^{\prime }=u_{t}{}^{\circ}p_{0}$ and manually-tracked *p*_*t*_ landmarks, defined by

(7) $$EPE\left(p,p^{\prime }\right)=\;\sqrt{{\left(p_{x}-p_{x}{}^{\prime }\right)}^{2}\;+\;{\left(p_{y}-p_{y}{}^{\prime }\right)}^{2}.}$$

Due to temporal misalignment between the tagging and cine acquisitions, EPE was evaluated only at ES (*t* = *t*_*ES*_). Specifically, let *p*_*ij*_(*t*) denote the manually-tracked landmarks of subject *i* at frame *t* by observer *j*. The accuracy of CarMEN was assessed using the average EPE

(8) $$\begin{array}{r} AEPE=\frac{1}{2n}\overset{n}{\underset{i=1}{\sum }}\overset{2}{\underset{j=1}{\sum }}EPE\left(p_{ij}\left(t_{ES}\right),\;u_{i}\left(t_{ES}\right)∙p_{0}\right). \end{array}$$

Our results were compared to those reported by the four groups that responded to the challenge (42), MEVIS (46), IUCL (9), UPF (11), and INRIA (12, 47). All groups submitted tagging-based motion estimates, but only UPF and INRIA provided estimates based on cine-MRI.

#### Strain Validation and Intra-Scanner Repeatability

The tagging-MRI method with the lowest AEPE at ES was used as the reference for strain analysis. The tagging-MRI-based motion estimates were registered and resampled to the cine-MRI space. Global strain and SR values throughout the entire cardiac cycle were derived from the resampled estimates as described in (48). Global- and regional-based analyses were performed to assess the repeatability of measures from two acquisitions. Relative changes (RC) and absolute relative changes (aRC) were calculated, taking the first acquisition as the reference. ESS and SR were calculated for the global-based analysis, and for region-based analyses, ESS values were normalized using the AHA polar map, and both RC and aRC were evaluated for each of the segments in the polar map.

#### Statistics

For validation, Bland-Altman analysis was used to quantify agreement between predicted and tagging strain measures. We used the term *bias* to denote the mean difference and the term *precision* to denote the standard deviation of the differences, the latter computed with 1-degree of freedom. Differences were also assessed using a paired *t*-test with Bonferroni correction for multiple comparisons. For global- and regional-based analyses of strain intra-scanner repeatability, ICC estimates and their 95% confidence intervals (CI) were calculated based on a single-rating, absolute agreement, 2-way mixed-effects model. Analyses were performed on Python v3.4 with the statistical pingouin module (49).

## Results

### Segmentation and Motion Estimation

Centering, segmentation, and motion estimation for an entire cardiac cycle (~25 frames) was accomplished in <13 s on a 12GB GPU and <2.2 min on a 32 GB RAM CPU. VCN located the LV center of mass with a median error of 1.3 mm.

Training with a MDC loss function resulted in slightly more accurate segmentations compared to CCE (Supplementary Table 2), therefore the MDC-trained model was used for all remaining analyses. With this model, correlation of CarSON and manual LV volumetric measures was >0.98 across all measures (Table 1), and biases in EF (+0.25 ± 3.2%), ED (+0.76 ± 6.7 mL), and ES (+0.19 ± 5.8 mL) volumes, and mass (+1.4 ± 10.3 g) were not significant. Further, these biases were smaller than those obtained with other methods, which were positive for LV EDV (1.5–3.7 mL), negative for LVM (−2.1 to −2.9 g), and close to zero (±0.5%) for EF. Simantiris et al. (18) obtained the best precision for LV EF (2.7 vs. 3.2% variance with CarSON), EDV (4.6 vs. 6.7 mm), and LVM (6.5 vs. 10.3 g). Isensee et al. (39) obtained the best results on geometric metrics, i.e., lower HD for the LV (ED 5.5 vs. 5.7 mm; ES 6.9 vs. 7.7 mm) and LVM (7.0 vs. 8.1 mm; 7.3 vs. 9.2 mm), and higher DSC for the LVM (0.904 vs. 0.898; 0.923 vs. 0.913). The DSC for the LV was similar for all methods (~0.967, ~0.929). MAE for the LV EDV and LVM were 5.3 ± 4.1 mL and 6.8 ± 6.5 g.

**Table 1:** State-of-the-art methods for left-ventricular segmentation shown at end-diastole (ED) and end-systole (ES) on the ACDC test set compared to proposed approach.

**Left-ventricle label**		**Dice similarity coefficient**		**Hausdorff distance**		**Ejection fraction**			**End-diastolic volume**		
		**ED**	**ES**	**ED**	**ES**	**Corr**.	**bias** **±** **std**		**Corr**.	**bias** **±** **std**	
		**val**.	**val**.	**mm**	**mm**	**val**.	**%**	**%**	**val**.	**mL**	**mL**
^*^	**CarSON**	0.967	0.929	5.656	7.676	0.990	0.252	3.183	0.996	0.762	6.672
1	Dong et al. (17)	0.967	0.928	6.366	7.573	0.993	−0.360	2.689	0.998	2.032	4.611
2	Simantiris and Tziritas (18)	0.967	0.928	5.476	6.921	0.991	0.490	2.965	0.997	1.530	5.736
3	Isensee et al. (19)	0.964	0.912	6.180	8.386	0.990	−0.476	3.114	0.997	3.746	5.146
**Myocardium label**		**Dice similarity coefficient**		**Hausdorff distance**		**Left-ventricular mass**			**End-systolic volume**		
		**ED**	**ES**	**ED**	**ES**	**Corr**.	**bias** **±** **std**		**Corr**.	**bias** **±** **std**	
		**val**.	**val**.	**mm**	**mm**	**val**.	**g**	**g**	**val**.	**mL**	**mL**
^*^	**CarSON**	0.898	0.913	8.128	9.189	0.981	1.405	10.32	0.985	1.152	9.391
1	Dong et al. (17)	0.904	0.923	7.014	7.328	0.987	−2.547	8.28	0.988	−1.984	8.335
2	Simantiris and Tziritas (18)	0.891	0.904	8.264	9.575	0.992	−2.904	6.46	0.983	−2.134	10.11
3	Zotti et al. (20)	0.873	0.895	8.197	8.318	0.989	−2.1	7.91	0.988	−1.79	8.575

*Red are the best results for each metric*.

* *Proposed segmentation method*.

**Figure 5:**
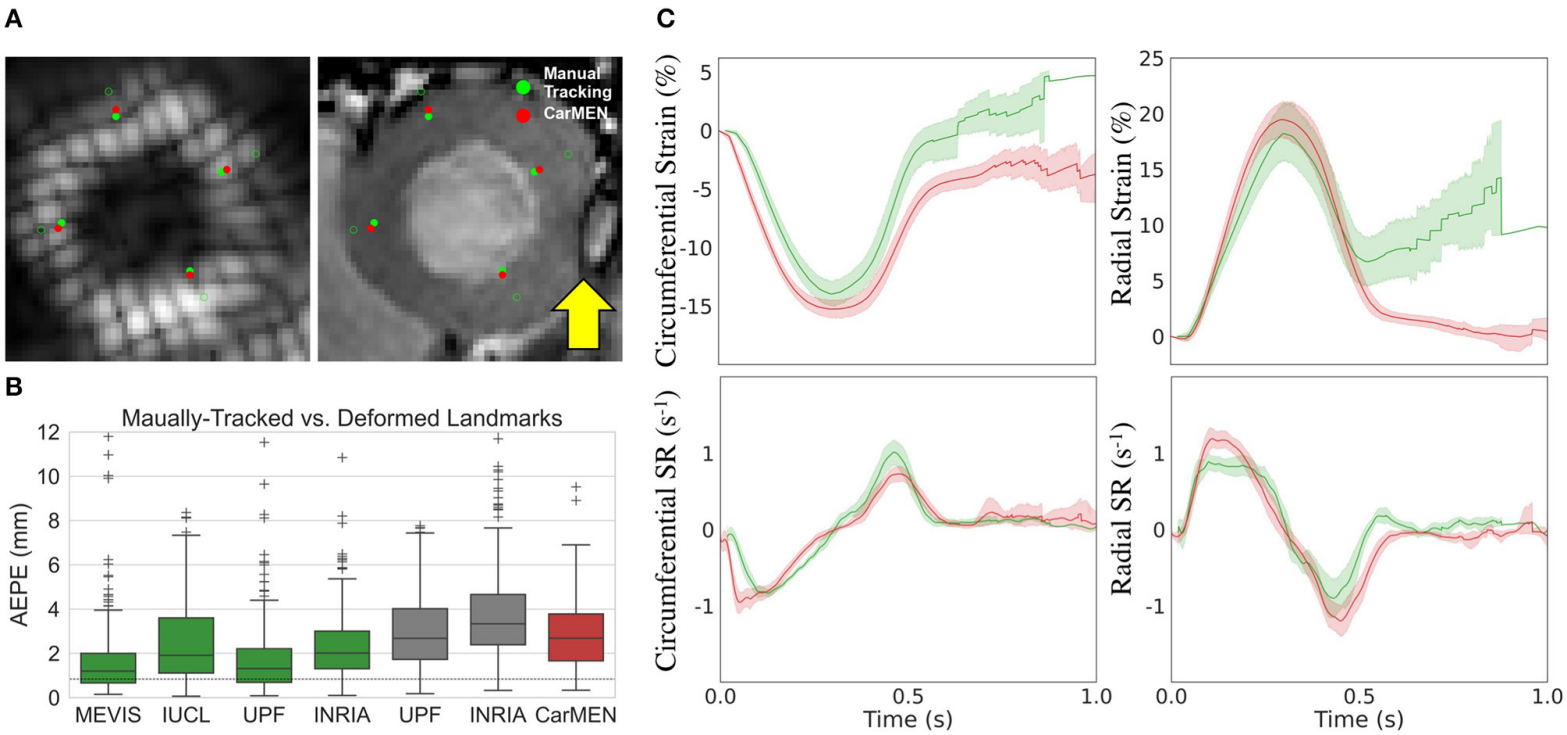
Validation of motion and strain using the CMAC dataset. **(A)** Landmarks at end-diastole (unfilled green) are manually-tracked (green) and deformed with CarMEN to end systole (red). Yellow arrow indicates a banding artifact. **(B)** Average end-point-error (AEPE) at end-systole between manual and CarMEN-deformed landmarks was assessed and compared to other methods. **(C)** MEVIS- (green) and DeepStrain-based (red) strain (top) and strain rate (SR, bottom) measures are compared.

Figure 5A illustrates a representative example of the tagging and cine images from a CMAC subject. Landmarks defined at ED were deformed to ES using the CarMEN estimates and compared to manual tracking. Banding artifacts on cine images showed no clear effect on derived motion estimates or landmark deformation, as shown in ES (Figure 5A, yellow arrow) or throughout the whole cardiac cycle (see Supplementary Video 1). The manual tracking inter-observer variability was 0.86 mm (Figure 5B, dotted line). Within cine-based techniques, CarMEN (2.89 ± 1.52 mm) and UPF (2.94 ± 1.64 mm) had lower (*p* <0.001) AEPE relative to INRIA (3.78 ± 2.08 mm), but there was no significant difference between CarMEN and UPF. All tagging-based methods had lower AEPE compared to cine approaches, particularly MEVIS (1.58 ± 1.45 mm). Finally, we evaluated the AEPE of the motion vectors in 10 synthetic datasets to compare our results against our previous CarMEN implementation. The AEPE was 1.6 ± 0.1 mm (1.1 ± 0.4 pixels) at ED, 2.1 ± 0.1 mm (1.33 ± 0.03 pixels) at ES, and 1.8 ± 0.2 mm (1.20 ± 0.2 pixels) combined.

### Strain Analysis

Table 2 shows the normal ranges (mean [95% CI]) of strain derived from cine-MRI data for all healthy subjects, including subjects from the training, validation, and repeatability cohorts. Across datasets, DeepStrain generated values with narrow CI of ESS (circumferential: 1.1%, radial: 2.5%), SRs (0.13 s^−1^, 0.19 s^−1^), and SRe (0.14 s^−1^, 0.26 s^−1^). Specifically, circumferential and radial values across datasets were: −16.9% [−17.4 −16.3] and 23.2% [22 24.4] for ESS, −1.1 s^−1^ [−1.2 −1.1] and 1.4 s^−1^ [1.3 1.5] for SRs, and 0.80 s^−1^ [0.73 0.86] and −1.5 s^−1^ [−1.6 −1.3] for SRe, accordingly. These values were similar to tagging-based ones, although circumferential SRe from cine-MRI data was lower, mostly in the train set (0.7 ± 0.2 s^−1^).

**Table 2:** Normal ranges of strain with DeepStrain in healthy subjects.

	**ACDC (*n* = 20)**	**CMAC (** ***n*** **=** **15)**		**MARTINOS (** ***n*** **=** **10)**		**COMBINED (*n* = 45)**
	**Cine**	**Tagging vs. Cine**		**Cine ACQ 1 vs. ACQ 2**		**Cine**
**End-systolic strain (%)**						
Circumferential	−17.8 (1.6)	−14.2 (2.2)	−15.3 (1.5)	−17.3 (0.7)	−17.5 (0.9)	−16.9 [−17.4 −16.3]
Radial	24.5 (2.9)	18.4 (5.1)	19.7 (3.4)	25.9 (3.4)	25.7 (4.1)	23.2 [22.0 24.4]
**Systolic strain rate (s** ^**−1**^ **)**						
Circumferential	−1.1 (0.2)	−0.9 (0.1)	−1.2 (0.2)	−1.0 (0.2)	−1.0 (0.2)	−1.1 [−1.2 −1.1]
Radial	1.3 (0.4)	1.0 (0.2)	1.3 (0.2)	1.7 (0.3)	1.6 (0.3)	1.4 [1.3 1.5]
**Early-diastolic strain rate (s** ^**−1**^ **)**						
Circumferential	0.7 (0.2)	1.2 (0.2)	0.8 (0.1)	1.0 (0.2)	1.0 (0.2)	0.80 [0.73 0.86]
Radial	−1.4 (0.5)	−1.2 (0.5)	−1.4 (0.3)	−1.8 (0.3)	−1.7 (0.4)	−1.5 [−1.6 −1.3]

*Tagging-based measures are shown for the CMAC cohort. DeepStrain repeatability is shown for two acquisitions (ACQ). MEVIS was used to calculate tagging measures. Data are presented as mean (standard deviation), and as mean [95% confidence interval] for all three datasets combined*.

Comparison of tagging- and cine-based strain measures with matched subjects showed an overall agreement in timing and magnitude of strain and SR throughout the cardiac cycle, although there were visual differences in peak SR parameters (Figure 5C). Visual inspection of image artifacts on cine data showed no evidence that these artifacts affected strain values derived with DeepStrain (Supplementary Figure 1). Quantitative comparisons of tagging- and cine-based measures showed biases in circumferential ESS (−14.2 ± 2.2 vs. −15.3 ± 1.5%; bias −1.17 ± 2.93%), radial ESS (18.4 ± 5.1 vs. 19.7 ± 3.4%; +1.26 ± 5.37%), and SRe (−1.2 ± 0.5 vs. −1.4 ± 0.3; −0.21 ± 0.52 s^−1^) were not significantly different from zero (Supplementary Figure 2). However, there were larger differences (*p* < 0.01) in radial SRs (1.0 ± 0.2 vs. 1.3 ± 0.2 s^−1^; 0.32 ± 0.34 s^−1^), and circumferential SRs (−0.9 ± 0.1 vs. −1.2 ± 0.2 s^−1^; 0.30 ± 0.22 s^−1^) and SRe (1.2 ± 0.2 vs. 0.8 ± 0.1 s^−1^; 0.40 ± 0.23 s^−1^).

**Figure 6:**
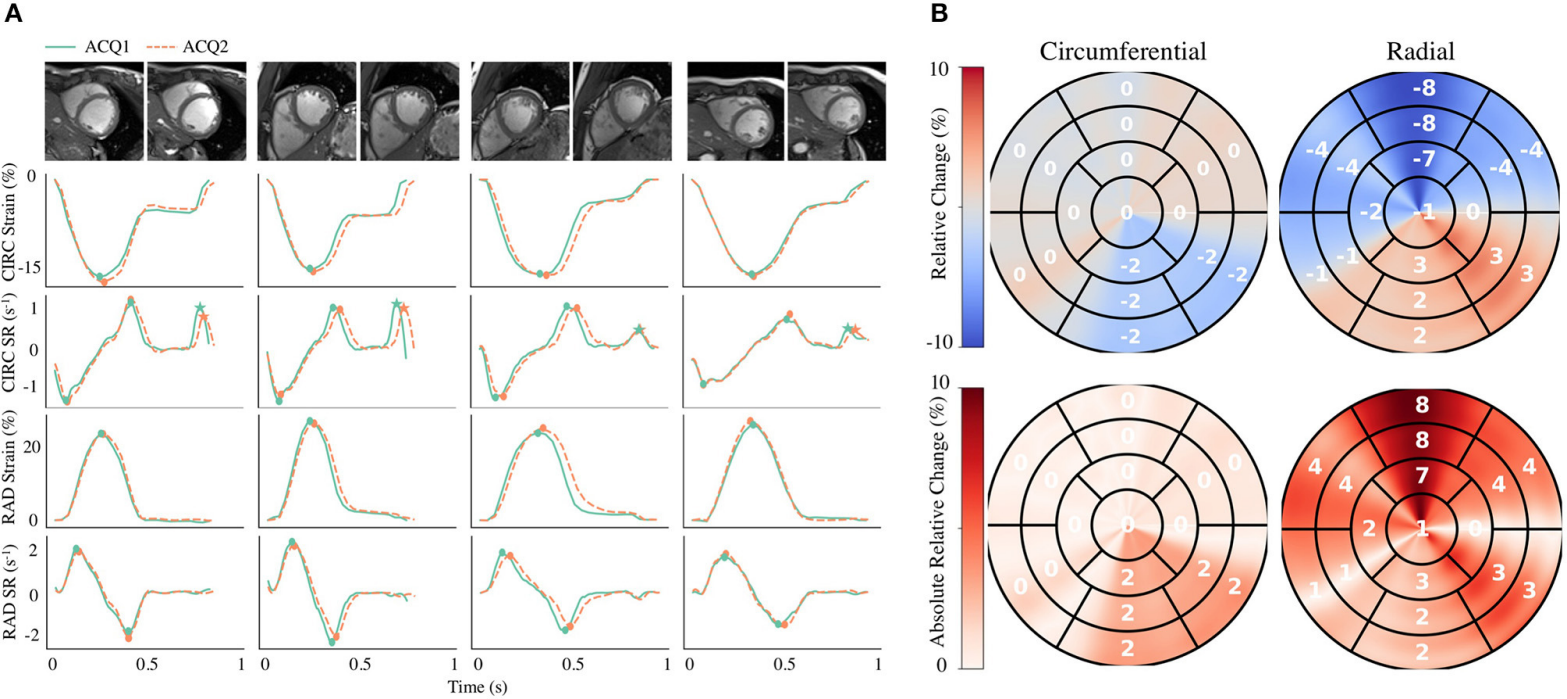
Intra-scanner repeatability of global and regional myocardial strain measures on the MARTINOS dataset. **(A)** Circumferential (CIRC) and radial (RAD) strain and strain rate (SR) curves across time derived from two different acquisitions (ACQ). Four representative healthy subjects are shown, including the correspond cine images used for analyses. **(B)** Polar maps for all subjects were used to evaluate the relative change and absolute relative change across polar map segments. Circles represent peak systolic and early-diastolic strain values. Stars represent peak late-diastolic strain values.

Global strain time series derived from repeated acquisitions are shown in Figure 6A. The overall bias in circumferential and radial ESS were 0.17 and −0.16%, accordantly. Average RC between parameters was less than ±1% for ESS and less than ±5% for peak SR (Table 3). Average aRC was ~5% for ESS (circumferential: 3.0 ± 2.0%; radial: 5.1 ± 5.8%), ~8% for SRs (8.0 ± 6.8%; 7.7 ± 4.0%), and ~10% for SRe (10.2 ± 7.8%; 9.2 ± 8.6%). Mean ICC values showed repeatability was good to excellent for ESS (0.75; 0.90), SRs (0.77, 0.91), and SRe (0.83, 0.84). The limits-of-agreement (LoA), which defines the interval where to find the expected differences in 95% of the cases assuming normally distributed data, were ~2 and ~6% for circumferential and radial ESS, and ~0.5 s^−1^ for SR measures. Average RC and aRC across regional segments were within ±2% for circumferential and ±5% for radial ESS, except in anterior segments (±8%) radially (Figure 6B). Regional mean ICC values showed good to excellent repeatability across all segments, except circumferentially near inferoseptal, inferior, and inferolateral walls were repeatability was moderate (Supplementary Table 3). LoAs showed that 95% of differences occurred within ~5 and ~10% intervals for circumferential and radial ESS.

**Table 3:** Intra-scanner repeatability of global circumferential and radial end-systolic strain (ESS) measures.

**Measure**	**RC (%)**	**aRC (%)**	**ICC [95% CI]**	**LoA**
Circumferential ESS	1.0 (3.6)	3.0 (2.0)	0.75 [0.22–0.92]	[−1.36 1.02%]
Radial ESS	−0.9 (7.9)	5.1 (5.8)	0.90 [0.64–0.97]	[−3.03 3.36%]
Circumferential SRs	0.8 (10.8)	8.0 (6.8)	0.77 [0.31–0.94]	[−0.23 0.22 s^−1^]
Radial SRs	−4.9 (7.4)	7.7 (4.0)	0.91 [0.67–0.98]	[−0.15 0.34 s^−1^]
Circumferential SRe	2.5 (13.0)	10.2 (7.8)	0.83 [0.47–0.96]	[−0.26 0.22 s^−1^]
Radial SRe	−2.5 (12.7)	9.2 (8.6)	0.84 [0.50–0.96]	[−0.32 0.41 s^−1^]

### Evaluation in Patients With Cardiovascular Disease

**Figure 7:**
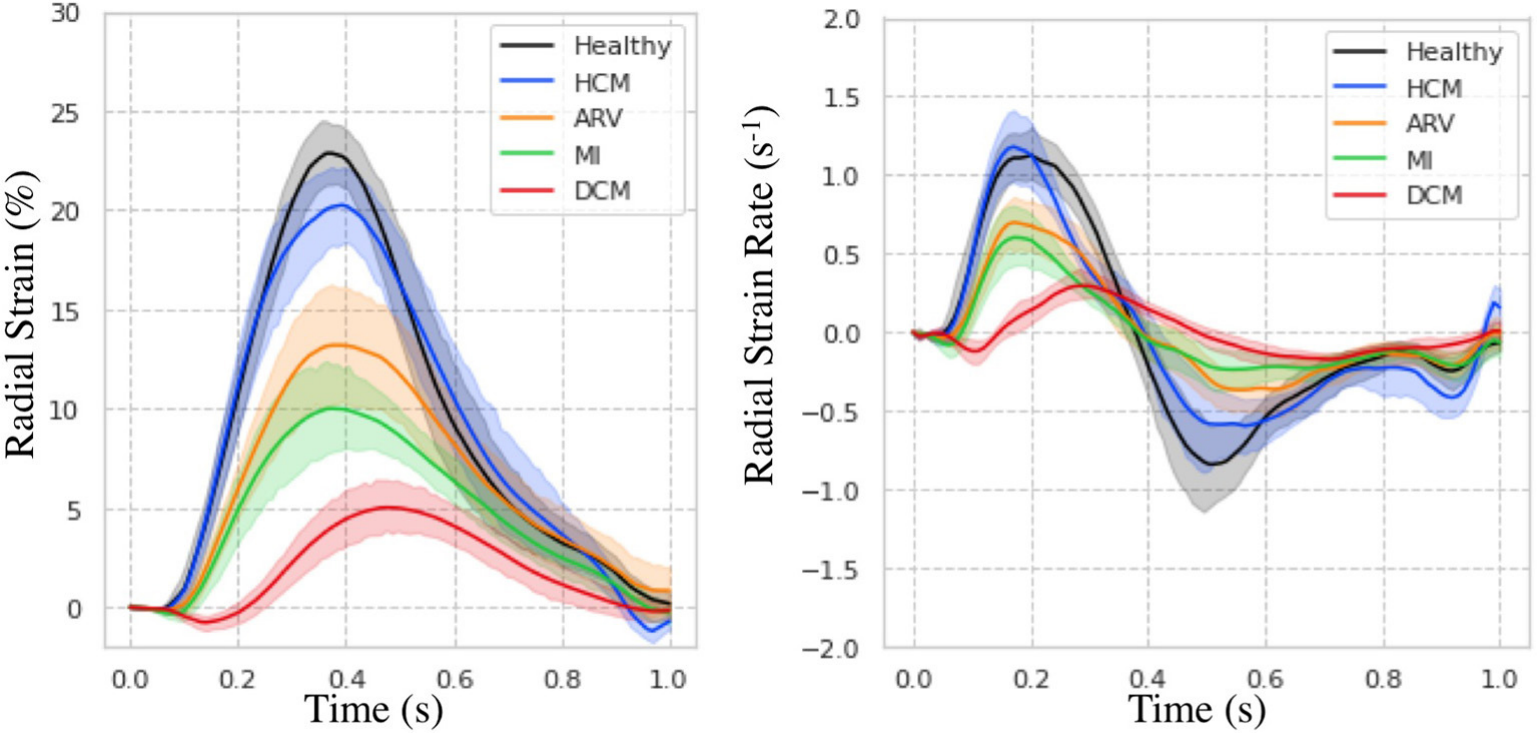
Strain measures on the ACDC train set. Radial strain **(Left)** and strain rate **(Right)** across time is shown for healthy subjects and patients with hypertrophic cardiomyopathy (HCM), abnormal right ventricle (ARV), myocardial infarction (MI), and dilated cardiomyopathy (DCM).

**Figure 8:**
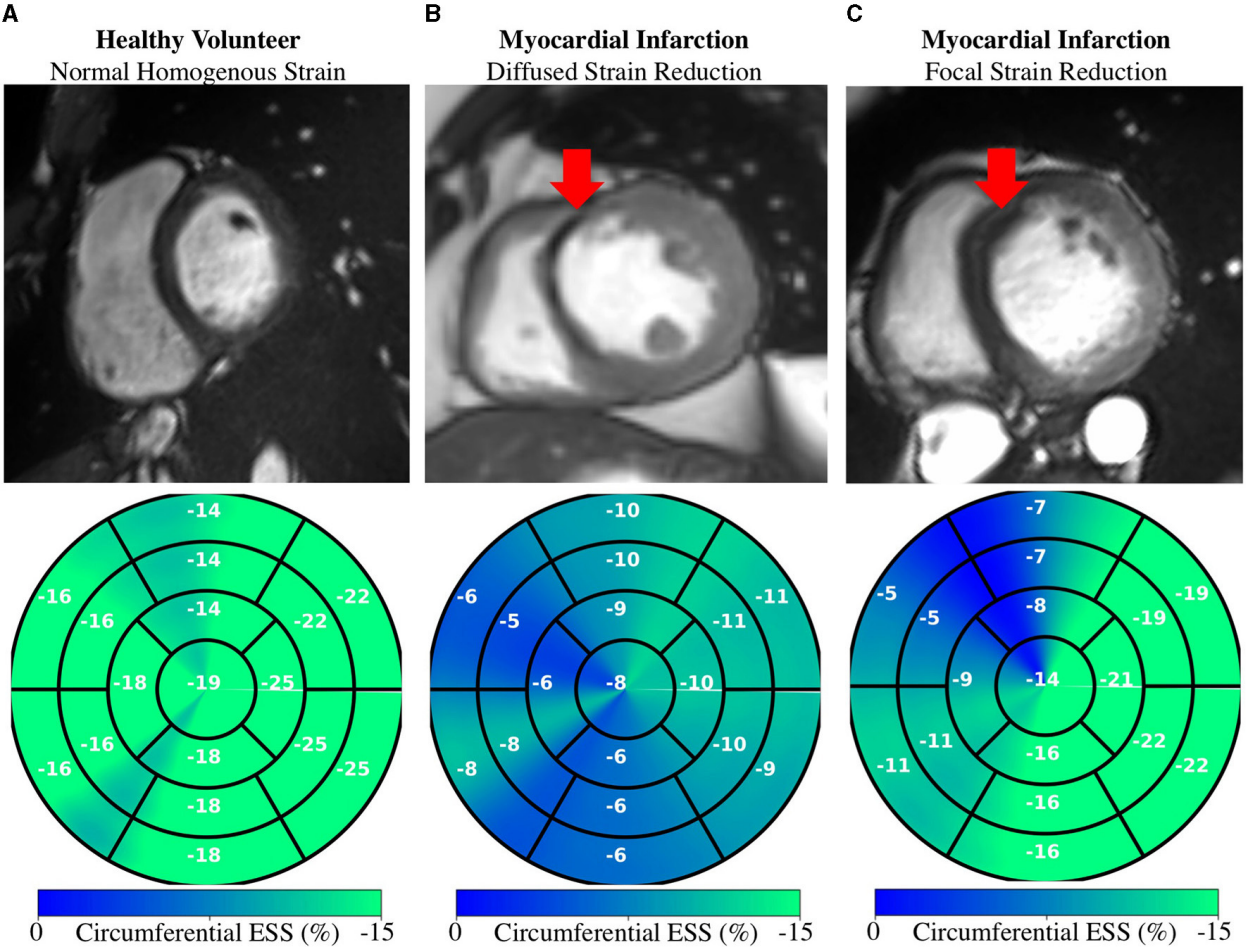
Regional Strain: Diffused vs. focal abnormalities. Anatomical (top) and regional (bottom) circumferential end-systolic strain (ESS) for healthy and MI subjects. **(A)** Healthy strain is homogenously distributed. **(B)** MI subject shows diffused strain reduction with an MI in the anteroseptal region. **(C)** Different MI subject shows a focal decrease in the anteroseptal region co-localized with the infarcted region (red arrows). MI, myocardial infarction.

Regional measures of ESS averaged over patient population (Supplementary Figure 3), as well as global values of strain and SR across the cardiac cycle (Figure 7) for all 100 subjects in the ACDC train set showed progressive decline in strain values starting with HCM, followed by ARV, MI, and DCM. Specifically, relative to the healthy group, radial ESS was reduced in all patient populations. Radial systolic and early-diastolic SR were also reduced in all patient groups, except for systolic SR in HCM. Figure 8 shows both the cine-MRI image and the circumferential ESS polar map of a healthy subject and two patients with MI. Strain values in the healthy polar map have a homogeneous distribution. In contrast, in one MI patient the map indicates a diffused reduction, and inspection of the myocardium on the cine-MRI image shows an anteroseptal infarct that coincides in location with segments with more prominent decreases in strain. In a different MI patient with an infarct located in a similar septal region, strain changes are focal and localized to the anteroseptal wall.

## Discussion

In this study we developed a fast DL framework for strain analysis based on cine-MRI data that does not make assumptions about the underlying physiology, and we benchmarked its segmentation, motion, and strain estimation components against the state-of-the-art. We compared our segmentations to other DL methods, motion estimates to other non-learning techniques, and strain measures to a reference tagging-MRI technique. We also presented the intra-scanner repeatability of DeepStrain-based global and regional strain measures, and showed that these measures were robust to image artifacts in some cases. Global and regional applications were also presented to demonstrate the potential clinical utilization of our approach. Our work is the first to report within a single study the characterization, validation, and repeatability of a learning-based method for strain analysis.

### Volumetric Measures

Segmentation from MRI data is a task particularly well-suited for CNNs given the excellent soft-tissue contrast, thus all top performing methods on the ACDC test set were based on DL approaches. Isensee et al. (39) had remarkable success on geometric metrics, but this and other approaches result in a systematic overestimation of the LV EDV and thus underestimation of LVM. In contrast, CarSON generated less biased measures of LV volumes and mass, which were not significant. Although Simantiris and Tziritas (18) obtained the most precise measures, possibly due to their extensive use of augmentation using image intensity transformations, across methods the precision of EF was within the ~3–5% (50) needed when it is used as an index of LV function in clinical trials (51). Lastly, we showed that the error in our measures of LV EDV and LVM was almost half the inter-observer (~10.6 mL, 12.0 g), and comparable to the intra-observer (~4.6 mL, 6.2 g) MAE reported in (41), but further investigations are required to assess the performance on more heterogeneous populations. Lastly, CarSON tends to perform better on DSC metrics compared to HD. This is mainly due to inclusion or exclusion of myocardium labels in most basal slides as described by Bernard et al. (41). However, the smoothing penalty used to train CarMEN reduces the impact on strain estimates by promoting smooth motion values across the myocardial tissue.

### Strain Validation

The application of myocardial strain to quantify abnormal deformation in disease requires accurate definition of normal ranges. However, previously reported normal ranges vary largely between modalities and techniques, particularly for radial ESS (4). In this study we showed DeepStrain generated strain measures with narrow CI in healthy subjects from across three different datasets. Although direct comparison with the literature is difficult due to differences in the datasets, overall our strain measures agreed with several reported results. Specifically, circumferential strain is in agreement with studies in healthy participants based on tagging (−16.6%, *n* = 129) and speckle tracking echocardiography (−18%, *n* = 265) datasets (52, 53), as well a recently proposed (−16.7% basal, *n* = 386) tagging-based DL method (48). Our radial strain values are in agreement with some tagging-based studies (26.5%, *n* = 129; 23.8% basal, *n* = 386) (48, 52), but are lower than most reported values (4). This is a result of smoothing regularization used during training to prevent overfitting. However, lowering the regularization without increasing the size of the training set would lead to increased EPE and wider CI. SR measures derived with DeepStrain were also in good agreement with previous tagging-based studies (52).

The CMAC dataset enabled us to compare our results to non-learning methods using a common dataset. We found that AEPE at ES was lower with tagging-based techniques, reflecting the advantage of estimating cardiac motion from a grid of intrinsic tissue markers (i.e., grid tagging lines). Further, the tagging techniques also benefited from the fact that landmarks were placed near the center of the myocardial wall borders, whereas motion estimation from tagging data at the myocardial walls and in thin-walled regions of the LV is less accurate due to the spatial resolution of the tagging grid (4). In addition, some of the tagging-MRI images did not enclose the whole myocardium and some contained imaging artifacts, which resulted in strain artifacts toward the end of the cardiac cycle. Nevertheless, MEVIS-based motion estimates achieved the lowest AEPE at ES and thus represent a reliable reference for end-systolic strain measures. This performance could be a result of their image term (4) that penalizes phase shifts in the Fourier domain instead of intensity values, an approach that is less affected by desaturation. The UPF approach also achieved a low AEPE using multimodal integration and 4D tracking to leverage the strengths of both modalities and improve temporal consistency (11). Specific differences in motion and strain measures between MEVIS and other techniques were thoroughly discussed by Tobon-Gomez et al. (42).

Using MEVIS as the tagging reference standard, we found no significant differences in measures of circumferential of radial and ESS. Validation studies have shown similar [±1%, (54–56)] or worse [±11% for radial, (55)] biases between cine feature tracking and tagging strain. However, these methods required manual contouring by an expert, whereas our method is fully-automatic. We found significant differences in SR measures between the two techniques that could be due to drift errors in the MEVIS implementation, i.e., errors that accumulate in sequential implementations in which motion is estimated frame-by-frame (42).

The AEPE on the synthetic dataset of 1.20 pixels was lower than our previously reported 1.7 pixels, which is expected as our previous implementation was not anatomically constrained. Although we did not observe considerable improvements in AEPE compared to tagging- and cine-based methods, an important advantage of our learning-based approach is the reduced computational complexity (~13 s in GPU) relative to the proposed MEVIS (1–2 h), IUCL (3–6 h), UPF (6 h), and INRIA (5 h) approaches (42). Specifically, because once trained our network does not optimize for a specific test subject (i.e., it does not iterate on the cine-data to generate the desired output), centering, segmentation, and motion estimation for the entire cardiac cycle can be accomplished much faster (<2 min in CPU). In addition, DeepStrain was trained on a relatively small dataset and was evaluated on data from different institutions and vendors, therefore its accuracy relative to non-learning methods could substantially improve through training with larger cohorts or application of data shift correction strategies. Furthermore, a joint optimization of segmentation and motion estimation CNNs could potentially improve the robustness of the workflow to undersampled data (24).

### Strain Repeatability

In this study we also evaluated the intra-scanner repeatability of strain measures in 10 healthy subjects, an important aspect to consider when assessing the potential clinical utility of DeepStrain. Confidence intervals in circumferential and radial ESS were 0 ± 1% and 0 ± 3%, better than the intra-observer variability reported using feature tracking in 10 healthy adults (57). A more recent study in 100 healthy individuals reported intra- and inter-observer repeatability for circumferential (ICC intra: 0.88, ICC inter: 0.88) and radial ESS (0.82, 0.79), which were comparable to our results for circumferential ESS (0.75) and radial ESS (0.90) using only 10 subjects. Finally, our repeatability of SR measures was good to excellent, similar to that reported for healthy (*n* = 20) and patient (*n* = 60) populations (58). Thus, without requiring expert operators, DeepStrain achieved better or equal repeatability compared to feature tracking methods.

### Potential Clinical Applications

DeepStrain could be applied in a wide range of clinical applications, e.g., automated extraction of imaging phenotypes from large-scale databases (59). Such phenotypes include global and regional strain, which are important measures in the setting of existing dysfunction with preserved EF (3). DeepStrain generated measures of global strain and SR over the entire cardiac cycle from a cohort of 100 subjects in <2 min. These results showed that radial SRe was reduced in patients with HCM and ARV, despite having a normal or increased LV EF. Decreased SRe with normal EF is suggestive of subclinical LV diastolic dysfunction, which is in agreement with previous findings (60, 61). Our results also showed DeepStrain-based maps could be used to characterize regional differences between groups.

At an individual level, we showed that in MI patients, polar segments with decreased circumferential strain matched myocardial regions with infarcted tissue. Further, we showed that the changes in regional strain due to MI can be both diffuse and focal. These abnormalities could be used to discriminate dysfunctional from functional myocardium (62), or as inputs for downstream classification algorithms (63). More generally, DeepStrain could be used to extract interpretable features (e.g., strain and SR) for DL diagnostic algorithms (64), which would make understanding of the pathophysiological basis of classification more attainable (65).

### Study Limitations

A limitation of our study was the absence of important patient information (e.g., age), which would be needed for a more complete interpretation of our strain analysis results, for example to assess the differences in strain values found between the healthy subjects from the ACDC and CMAC datasets. Nevertheless, using publicly available data enables the scientific community to more easily reproduce our findings, and compare our results to other techniques. Another limitation was the absence of longitudinal analyses, i.e., longitudinal strain was not reported because it is normally derived from long-axis cine-MRI data not available in the training dataset. The size of the datasets is another potential limitation. The number of patients used for training is much smaller than the number of trainable parameters, potentially resulting in some degree of overfitting. To correct this, the training set for motion estimation could be expanded by validating the proposed segmentation network on more heterogeneous populations. The use of strain minimization deviation as a training heuristic also serves as a learning constrain but has not been validated, and could potentially prevent identification of subtle disease due to loss of sensitivity to abnormal strain. While our repeatability results were promising despite testing in only a small number of subjects, repeatability in patient populations was not shown. Further, reproducibility across sites and vendors was not assessed. In addition, the accuracy of the motion estimates on patient populations with regional dysfunction was not assessed, and we did not quantify the effect of dataset shift errors that might occur when applying our method to new datasets.

### Conclusion

We developed an end-to-end learning-based workflow for strain analysis that is fast, operator-independent, and leverages real-world data instead of making explicit assumptions about myocardial tissue properties or geometry. This approach enabled us to derive strain measures from new data that were repeatable, and comparable to those derive from dedicated tagging data. These technical and practical attributes position DeepStrain as an excellent candidate for use in routine clinical studies or data-driven research.

## Data Availability Statement

The datasets presented in this study can be found in online repositories. The names of the repository/repositories and accession number(s) can be found at: https://github.com/moralesq.

## Ethics Statement

The studies involving human participants were reviewed and approved by written consent was obtaining from all volunteers with approval of the institutional review board (2018P002912) and in agreement with the Health Insurance Portability and Accountability Act (HIPAA) at the Massachusetts General Hospital. The patients/participants provided their written informed consent to participate in this study.

## Author Contributions

MM designed the workflow, performed data analysis, and drafted the manuscript. All other authors revised the drafted manuscript and contributed critical intellectual content. This manuscript has been revised and approved by all authors.

## Conflict of Interest

The authors declare that the research was conducted in the absence of any commercial or financial relationships that could be construed as a potential conflict of interest.

## Publisher's Note

All claims expressed in this article are solely those of the authors and do not necessarily represent those of their affiliated organizations, or those of the publisher, the editors and the reviewers. Any product that may be evaluated in this article, or claim that may be made by its manufacturer, is not guaranteed or endorsed by the publisher.

## Abbreviations

ACDC: automated cardiac diagnosis challenge
AHA: American heart association
ARV: abnormal right ventricle
CCN: categorical cross-entropy
CMAC: cardiac motion analysis challenge
CarMEN: cardiac motion estimation network
CarSON: cardiac segmentation network
CNN: convolutional neural network
DCM: dilated cardiomyopathy
DL: deep learning
ED: end-diastole
EF: ejection fraction
EPE: end-point error
ES: end-systole
ESS: end-systolic strain
HCM: hypertrophic cardiomyopathy
LV: left-ventricular
LVM: left-ventricular myocardium
MDC: multi-class Dice coefficient
MI: myocardial infarction
MRI: magnetic resonance imaging
MRXCAT: magnetic resonance-extended cardiac-torso
RC: relative change
RV: right-ventricular
SR: strain rate
SRe: early-diastolic strain rate
SRs: systolic strain rate
VCN: ventricular centering network.
