# Supplementary material for: DeepStrain: A Deep Learning Workflow for the Automated Characterization of Cardiac Mechanics
Source: Front Cardiovasc Med. 2021 Sep 3;8:730316. doi: 10.3389/fcvm.2021.730316 (PMC8446607; doi:10.3389/fcvm.2021.730316)
Supplement: Supplementary file 2 [file Data_Sheet_1.PDF]

Supplementary Sections

S1. Acquisition Protocol

Subjects were recruited to undergo repeated scans. Standard cardiac MRI protocols were acquired on a 3T MRI system (Biograph mMR, Siemens Healthiness, Germany, Erlangen) in 10 healthy volunteers during two separate consecutive scan sessions on the same day. Written consent was obtaining from all volunteers with approval of the institutional review board (2018P002912) and in agreement with the Health Insurance Portability and Accountability Act (HIPAA) at the Massachusetts General Hospital. The major inclusion criteria were >21 years old, no history of cardiovascular diseases, and no MR contradictions. Each MRI protocol included standard localizer scans and a single slice cine acquisition in a long cardiac axis view, followed by a stack of short axis cines covering the heart from the base to the apex using a retrospectively ECG gated balanced steady-state gradient echo sequence (True FISP) with the following parameters; TR=38.61ms, TE=1.26ms, FA=28 degrees, FOV=277x340mm, matrix size=208x170, slice thickness=6mm, slice gap=6mm, FOV%=81.7%, bandwidth=925Hz with phase-encoding parallel imaging acceleration (PAT) of 2 and 25 cardiac frames over consecutive breath-holds. After each full protocol, the volunteers were asked to leave the scanner room before going back in for a second acquisition of exactly the same protocol.

S2. Green-Lagrange Strain and Shear

Following the mathematical conventions for nonlinear elastodynamics (Murea, 2017), let $\boldsymbol{u}(\mathbf{X}, t)$ denote the myocardial displacement from a fully-relaxed undeformed configuration at time $t = 0$ to a deformed configuration at $t > 0$, where $\mathbf{X}$ denotes the origin (i.e., reference) coordinate system. The partial derivative of the displacement $\boldsymbol{u}$ with respect to the material coordinates $\mathbf{X}$ is given by

$$\nabla_{\mathbf{X}}\boldsymbol{u} = \begin{pmatrix} \frac{\partial u_x}{\partial x} & \frac{\partial u_x}{\partial y} & \frac{\partial u_x}{\partial z} \\ \frac{\partial u_y}{\partial x} & \frac{\partial u_y}{\partial y} & \frac{\partial y}{\partial z} \\ \frac{\partial u_z}{\partial x} & \frac{\partial u_z}{\partial y} & \frac{\partial u_z}{\partial z} \end{pmatrix},$$

where u_x, u_y, and u_z denote the *x, y,* and *x* components of motion (i.e., channels 1, 2, and 3 of CarMEN output). The gradient was computed using second order accurate central differences. The Green-Lagrange strain tensor can be defined as

$$\mathbf{E} = \begin{pmatrix} \mathrm{E}_{xx} & \mathrm{E}_{xy} & \mathrm{E}_{xz} \\ \mathrm{E}_{yx} & \mathrm{E}_{yy} & \mathrm{E}_{yz} \\ \mathrm{E}_{zx} & \mathrm{E}_{zy} & \mathrm{E}_{zz} \end{pmatrix} = \frac{1}{2}((\nabla_{\mathbf{X}}\boldsymbol{u})^T + \nabla_{\mathbf{X}}\boldsymbol{u} + (\nabla_{\mathbf{X}}\boldsymbol{u})^T\nabla_{\mathbf{X}}\boldsymbol{u}).$$

By defining $\mathbf{F} = \nabla_{\mathbf{X}}\boldsymbol{u} + \mathbf{I}$, where $\mathbf{I}$ is the unit matrix, the Green-Lagrange strain tensor can be written more compactly as

$$\mathbf{E} = \frac{1}{2}\,(\mathbf{F}^{\mathrm{T}}\mathbf{F} - \mathbf{I}).$$

The cylindrical system was defined by the cardiac long axis (z-axis), with $\theta = 0$ along the anterior wall (x-axis) increasing counterclockwise towards the septal wall (y-axis) as shown in the main Fig. 1. The origin of the coordinate system was defined as the center of mass, the circumferential direction was defined perpendicular to the radial in the short-axis plane. The tensor $\mathbf{E}$ can be expressed in cylindrical coordinates using the transformation (Gonzales, 2008):

$$\begin{pmatrix} \mathrm{E}_{rr} & \mathrm{E}_{r\theta} & \mathrm{E}_{rz} \\ \mathrm{E}_{\theta r} & \mathrm{E}_{\theta\theta} & \mathrm{E}_{\theta z} \\ \mathrm{E}_{zr} & \mathrm{E}_{z\theta} & \mathrm{E}_{zz} \end{pmatrix} = \begin{pmatrix} \cos\theta & \sin\theta & 0 \\ -\sin\theta & \cos\theta & 0 \\ 0 & 0 & 1 \end{pmatrix} \begin{pmatrix} \mathrm{E}_{xx} & \mathrm{E}_{xy} & \mathrm{E}_{xz} \\ \mathrm{E}_{yx} & \mathrm{E}_{yy} & \mathrm{E}_{yz} \\ \mathrm{E}_{zx} & \mathrm{E}_{zy} & \mathrm{E}_{zz} \end{pmatrix} \begin{pmatrix} \cos\theta & -\sin\theta & 0 \\ \sin\theta & \cos\theta & 0 \\ 0 & 0 & 1 \end{pmatrix}$$

Radial and circumferential strain are given by the diagonal components of the tensor:

$$E_{rr} = \cos\theta\left(\mathrm{E}_{xx}\cos\theta + \mathrm{E}_{xy}\sin\theta\right) + \sin\theta\,(\mathrm{E}_{yx}\cos\theta + \mathrm{E}_{yy}\sin\theta)$$

$$E_{\theta\theta} = -\sin\theta\left(-\mathrm{E}_{xx}\sin\theta + \mathrm{E}_{xy}\cos\theta\right) + \cos\theta\,(-\mathrm{E}_{yx}\sin\theta + \mathrm{E}_{yy}\cos\theta)$$

The non-diagonal (shear) components describe the change of angles:

$$E_{r\theta} = E_{\theta r} = \cos\theta\left(-\mathrm{E}_{xx}\sin\theta + \mathrm{E}_{xy}\cos\theta\right) + \sin\theta\,(-\mathrm{E}_{yx}\sin\theta + \mathrm{E}_{yy}\cos\theta)$$

Although only radial and circumferential strain were analyzed in our study, shear is also implemented in our software package[1].

References:

Murea, C. M. Nonlinear elastodynamics. Stable numerical schemes for fluids, structures and their interactions. Chapter 5, 133-145.

Gonzales, O. Stuart A. M. A first course in continuum mechanics. Chapter 5, 127

[1] https://github.com/moralesq/DeepStrain/blob/main/utils/myocardial_strain.py

S3. Network Architectures

Let **Ck** denote a Convolution-BatchNorm-PReLU layer with k filters. **CDk** denotes a Upsampling-Convolution-BatchNorm-PReLU layer with upsampling applied using nearest-neighbor interpolation of stride 2x2x2. Unless specified, all convolutions are 3x3x3 spatial filters applied with 1x1x1 stride. An encoding layer **Ek** consists of a Ck layer followed by a second Ck layer with stride 2x2x2. A third Ck layer follows but without BatchNorm-PReLU. The output is the residual connection made by element-wise addition of the second Ck layer before BatchNorm-PReLU are applied, and the third Ck layer. A decoding layer **Dk** consists of a Ck layer with (1x1x1)-sized filters followed by CDk and Ck layers. Thus, the 2x2x2 strided convolution in Ek downsamples by a factor of 2, whereas the upsampling operation with stride 2x2x2 in Dk upsamples by a factor of 2 With this notation, the encoder-decoder architecture common to all three networks consists of

encoder: E64-E128-E256-E512-E512-E512-E512. **decoder**: D512-D512-D512-D256-D128-D64.

After the last layer in the decoder, a final CDk layer without BatchNorm -PReLU and with (1x1x1)-sized filters is applied to map to the number of output channels (k=1 for VCN, 4 for CarSON, and 3 for CarMEN). Exceptions to the rules above are: (1) CarSON consists of two-dimensional operations, i.e., convolutions are 3x3 spatial filters applied with 1x1 stride. (2) For CarMEN we experimented with another implementation where all filters and strides were of size 1 along the third dimension, motivated by the fact that subjects had variable resolution along that dimension.

Supplementary Tables

SUPPLEMENTARY TABLE I

EFFECT OF CARMEN ANATOMICAL REGULARIZATION FUNCTION AND LAYER OPERATION SIZE k_z ON HEALTHY MYOCARDIAL STRAIN. **RED** INDICATES METHODS WITH THE LOWEST STANDARD DEVIATION IN STRAIN.

**Anatomical Regularizer Loss Function**	Regularization Hyperparameters			k_z	Radial ESS (%)		Circumferential Ecc (%)	
	λ_i	λ_a	λ_s		mean	std	mean	std
Categorical cross-entropy	0.01	1.0	0.3	3	34	14	-15	5
Multiclass Dice	0.01	1.0	0.3	3	27	10	-16	3
Multiclass Dice	0.01	1.0	0.3	1	27	8	-16	3
Categorical cross-entropy	0.01	0.5	0.3	3	36	12	-15	3
Multiclass Dice	0.01	0.5	0.3	3	20	7	-16	4
Multiclass Dice	0.01	0.5	0.3	1	22	**7**	-17	**3**

SUPPLEMENTARY TABLE II

EFFECT OF CARSON LOSS FUNCTION ON LEFT-VENTRICULAR SEGMENTATIONS. MULTICLASS DICE COEFFICIENT (MDC) AND CATEGORICAL CROSS-ENTROPY (CCE) FUNCTIONS ARE COMPARED AT END-DIASTOLE (ED) AND END-SYSTOLE (ES) ON THE ACDC TEST SET. RED ARE THE BEST RESULTS FOR EACH METRIC.

**Left-Ventricle Label**	**Dice Similarity Coefficient**		**Hausdorff Distance**		**Ejection Fraction**			**End-Diastolic Volume**		
	ED	ES	ED	ES	Corr.	bias±std		Corr.	bias±std	
	val.	val.	mm	mm	val.	%	%	val.	mL	mL
CarSON-MDC	0.97	0.93	5.66	7.68	0.990	0.25	3.18	0.996	0.76	6.67
CarSON-CCE	0.95	0.89	6.87	8.68	0.990	0.88	3.13	0.996	0.85	6.72
**Myocardium Label**	**Dice Similarity Coefficient**		**Hausdorff Distance**		**Left-Ventricular Mass**			**End-Systolic Volume**		
CarSON Loss	ED	ES	ED	ES	Corr.	bias±std		Corr.	bias±std	
	val.	val.	mm	mm	val.	g	g	val.	mL	mL
CarSON-MDC	0.90	0.91	8.13	9.19	0.981	1.41	10.3	0.985	1.15	9.39
CarSON-CCE	0.86	0.87	8.90	10.35	0.984	0.96	9.6	0.997	-1.06	6.39

Supplementary Table IIIII
Intra-scanner repeatability of regional strain measures.

Region	circ ICC [95% CI]	circ LoA	rad ICC [95% CI]	rad LoA
Basal Anterior	0.94 [0.79-0.99]	[-1.6 1.5]	0.78 [0.33-0.94]	[-8.2 4.0]
Basal Anteroseptal	0.89 [0.62-0.97]	[-1.7 1.7]	0.94 [0.79-0.99]	[-5.5 3.2]
Basal Inferoseptal	0.57 [-0.05-0.87]	[-3.9 3.9]	0.91 [0.67-0.98]	[-5.4 4.4]
Basal Inferior	0.56 [-0.06-0.87]	[-3.1 4.2]	0.82 [0.44-0.95]	[-4.8 5.8]
Basal Inferolateral	0.73 [0.24-0.93]	[-2.3 3.3]	0.83 [0.45-0.95]	[-5.5 7.8]
Basal Anterolateral	0.96 [0.84-0.99]	[-1.2 0.9]	0.82 [0.44-0.95]	[-6.4 4.4]
Mid-Anterior	0.95 [0.83-0.99]	[-1.5 1.4]	0.79 [0.37-0.94]	[-7.8 3.8]
Mid-Anteroseptal	0.88 [0.60-0.97]	[-1.7 1.7]	0.95 [0.81-0.99]	[-5.0 2.8]
Mid-Inferoseptal	0.56 [-0.06-0.87]	[-3.9 3.9]	0.91 [0.68-0.98]	[-5.3 4.2]
Mid-Inferior	0.55 [-0.07-0.87]	[-3.1 4.1]	0.83 [0.46-0.95]	[-4.7 5.8]
Mid-Inferolateral	0.73 [0.23-0.93]	[-2.3 3.2]	0.85 [0.50-0.96]	[-5.1 7.6]
Mid-Anterolateral	0.96 [0.83-0.99]	[-1.2 0.9]	0.83 [0.47-0.96]	[-6.2 4.2]
Apical-Anterior	0.95 [0.83-0.99]	[-1.3 1.2]	0.80 [0.39-0.95]	[-6.9 3.5]
Apical-Septal	0.75 [0.26-0.93]	[-2.8 2.9]	0.94 [0.79-0.99]	[-4.6 3.0]
Apical Inferior	0.54 [-0.09-0.86]	[-3.0 3.9]	0.85 [0.50-0.96]	[-4.6 6.0]
Apical Lateral	0.91 [0.67-0.98]	[-1.6 1.9]	0.83 [0.47-0.96]	[-5.7 6.0]
Apical	0.66 [0.10-0.90]	[-1.7 2.0]	0.89 [0.63-0.97]	[-4.7 3.9]

Supplementary Figures

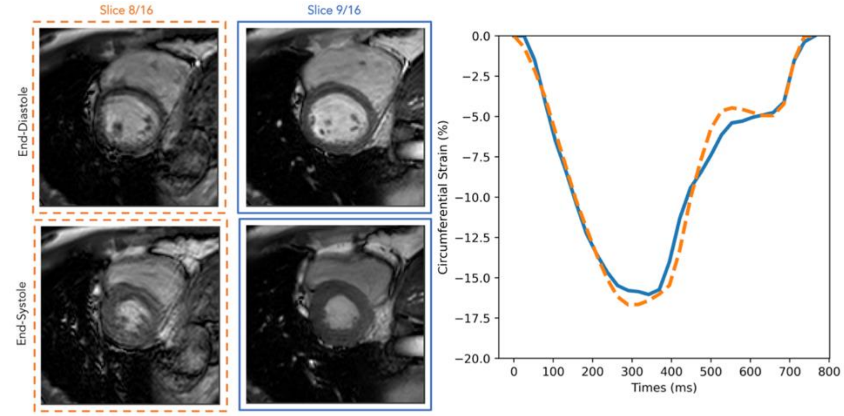

Fig. S1 Effects of image artifacts on myocardial strain estimates. In a healthy volunteer used to validate DeepStrain, imaging modality artifacts occurred during acquisition of slice 8 (left column), and were present at all time points (e.g., end-systole). Comparsion of the average strain, per-slice, against the adjacent slice without such artifacts (right column) showed little evidence that these artifacts affected the strain values derived with DeepStrain.

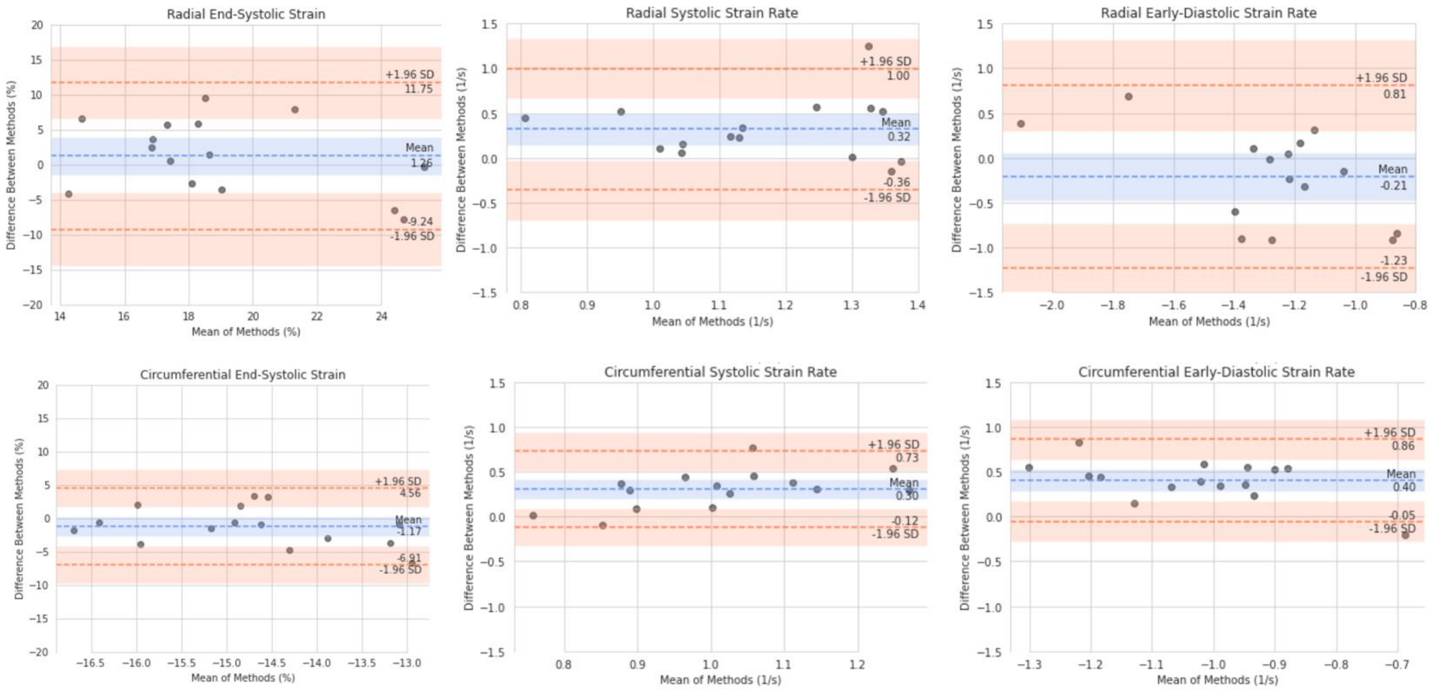

Fig. S2 Validation of myocardial strain estimates using tagging-MRI reference method. Bland-Altman plots of the strain measures obtained for the left ventricle at end-systole. The strain values obtained from DeepStrain were compared with those from the tagging reference standard. The first row shows the radial strain (first colum), systolic strain rate (second column), and early-diastolic strain rate (third column). The second row shows similar circumferential measures. Blue line denotes the mean difference; orange lines denote the 95% limits of agreement (mean ± 1.96 · standard devidation [SD]).

and strain rate intra-scanner repeatability.

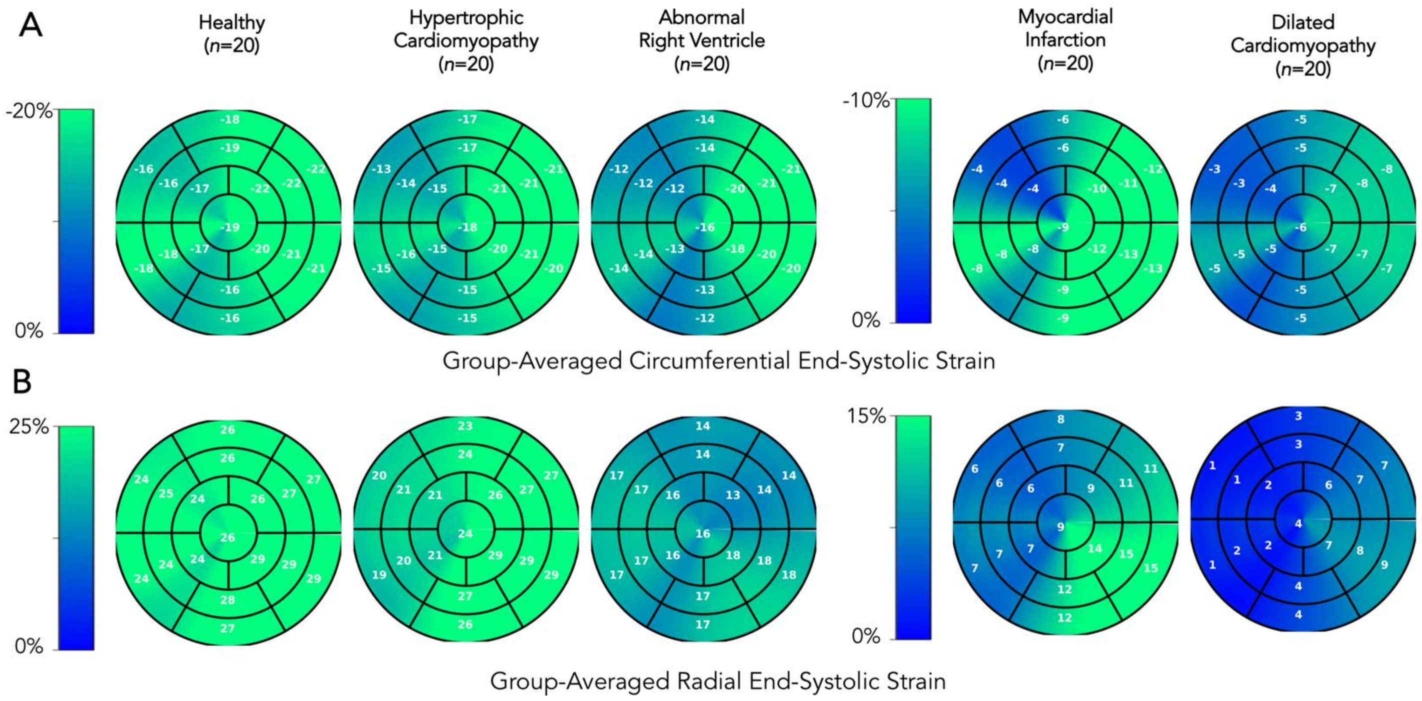

Fig. S3 Group-wise comparison, a potential clinical application of regional strain. Group-averaged regional strain values evaluated at end-systole for five groups shows progressive decline, starting with patients with hypertrophic cardiomyopathy (second column), and followed by patients with abnormal right ventricle, myocardial infarction, and dilated cardiomyopathy.
